# Pulmonary artery rupture by pulmonary artery catheter in cardiac surgery: a case report and review of literature

**DOI:** 10.3389/fcvm.2025.1567723

**Published:** 2025-04-28

**Authors:** Yuta Nakamura, Keita Saku, Suzu Homma, Yoko Midorikawa, Tsuyoshi Yamabe, Takashi Ota

**Affiliations:** ^1^ Department of Anesthesiology, Shonan Kamakura General Hospital, Kamakura, Japan; ^2^ Department of Cardiovascular Dynamics, National Cerebral and Cardiovascular Center Research Institute, Suita, Japan; ^3^ Department of Cardiovascular Surgery, Shonan Kamakura General Hospital, Kamakura, Japan

**Keywords:** cardiovascular anesthesia, pulmonary artery catheter, pulmonary artery rupture, aortic dissection, extracorporeal membrane oxygenation, coil embolization, intensive care

## Abstract

The pulmonary artery catheter (PAC) is widely used in cardiac surgery for monitoring hemodynamics and cardiovascular function. Complications including pulmonary artery injury causing massive intratracheal hemorrhage are rare but can be life-threatening. We report a case of intratracheal bleeding (3,000 ml) caused by PAC-induced pulmonary artery injury during cardiac surgery and after weaning from cardiopulmonary bypass (CPB). During surgery for acute type A aortic dissection followed by CPB weaning, pulsatile bleeding from the endotracheal tube and desaturation were observed. We reinstituted CPB and placed a right-sided double-lumen tube to compress the injured site of the lung and protect the contralateral site. Following initial bleeding control, we conducted coil embolization to treat tracheal obstruction by a pseudoaneurysm on day 7. A review of 21 recent cases of pulmonary artery injury during cardiac surgery showed that most cases occurred during CPB weaning, manifested hemoptysis, and were treated by coil embolization. This case underscores the importance of enhanced PAC monitoring even after CPB weaning and the need for prompt evaluation and intervention when pulmonary artery injury is suspected during cardiac surgery.

## 1 Introduction

The use of pulmonary artery catheter (PAC) in critical care, major non-cardiac surgeries, and acute coronary syndrome patients has been extensively studied. However, evidence has shown that PAC use in these settings does not improve outcomes and is associated with a higher incidence of complications (1, 2). In contrast, recent reports highlight the utility of PAC in the management of cardiogenic shock, particularly in the context of initiation and management of mechanical circulatory support (MCS) (3). Furthermore, PAC continues to be used in more than one-third of all cardiac surgeries (4, 5). Patients undergoing cardiac surgery often have severe cardiac diseases with increased risk of hemodynamic instability and cardiac dysfunction, and use of PAC is likely beneficial in this patient population. However, PAC has the risk of highly fatal complications such as pulmonary artery injury causing massive intratracheal hemorrhage, with a reported incidence of 0.1%–0.8% and mortality rate of 45%–60% (6, 7). Therefore, it is recommended to carefully select cases in which intraoperative PAC placement is likely to be beneficial.

We encountered a case of pulmonary artery injury associated with PAC use, resulting in intratracheal bleeding of approximately 3,000 ml, during surgery for acute type A aortic dissection followed by weaning from cardiopulmonary bypass (CPB). Comprehensive management including CPB reinstitution to stabilize hemodynamics and limit pulmonary blood flow, placement of a right-sided double-lumen tube to compress the injured site and protect the contralateral lung, and coil embolization for a pseudoaneurysm successfully resolved the condition without the need for pulmonary resection.

We also reviewed 21 recent cases of pulmonary artery injury during cardiac surgery to gain better understanding of PAC-induced pulmonary artery injury. This case and literature review underscore the importance of enhanced PAC monitoring even after CPB weaning and the need for prompt evaluation and intervention when pulmonary artery injury is suspected during cardiac surgery.

## 2 Case report

A 64-year-old man was transported to our emergency department due to sudden onset of chest and back pain at rest, followed by weakness and left-sided hemiparesis. He had hypertension managed with amlodipine, but no history of smoking, diabetes mellitus and cardiovascular diseases. On admission, his vital signs were stable except for blood pressure discrepancies. Right gaze deviation, dysarthria, and incomplete paralysis of the left upper and lower limbs were present. Transthoracic echocardiography showed no abnormalities in contraction, pericardial effusion, and aortic valve regurgitation. Contrast-enhanced computed tomography (CT) of the chest identified aortic dissection extending from the sinus of Valsalva to the common iliac arteries. The dissection was categorized as type A according to Stanford classification, type I according to DeBakey classification, and AE2M2 + M3- according to TEM classification. The diagnosis was acute type A aortic dissection requiring emergent total arch replacement with frozen elephant trunk placement.

Anesthesia was induced uneventfully with propofol, fentanyl, and rocuronium, and was followed by endotracheal intubation with a single-lumen tube. Anesthesia was maintained with continuous propofol infusion. Invasive blood pressure monitoring was established via bilateral radial arteries and the left dorsalis pedis artery. A PAC was placed via the right internal jugular vein under transesophageal echocardiography (TEE) guidance. The catheter was positioned 3 cm proximal to the wedge position at a depth of 55 cm without complications. The central venous pressure (CVP) and pulmonary artery pressure (PAP) waveforms were appropriate. Anesthesia was maintained without complications. CPB was established by inserting a 21-Fr arterial cannula into the left femoral artery, and 24-Fr and 32-Fr venous cannulas into the superior and inferior vena cava, respectively. After achieving high-moderate hypothermia at 26°C, circulatory arrest with selective cerebral perfusion was established. The procedure, including arch branch reconstruction, progressed smoothly up until the stage immediately prior to weaning from CPB.

### 2.1 Timeline

**Figure 1:**
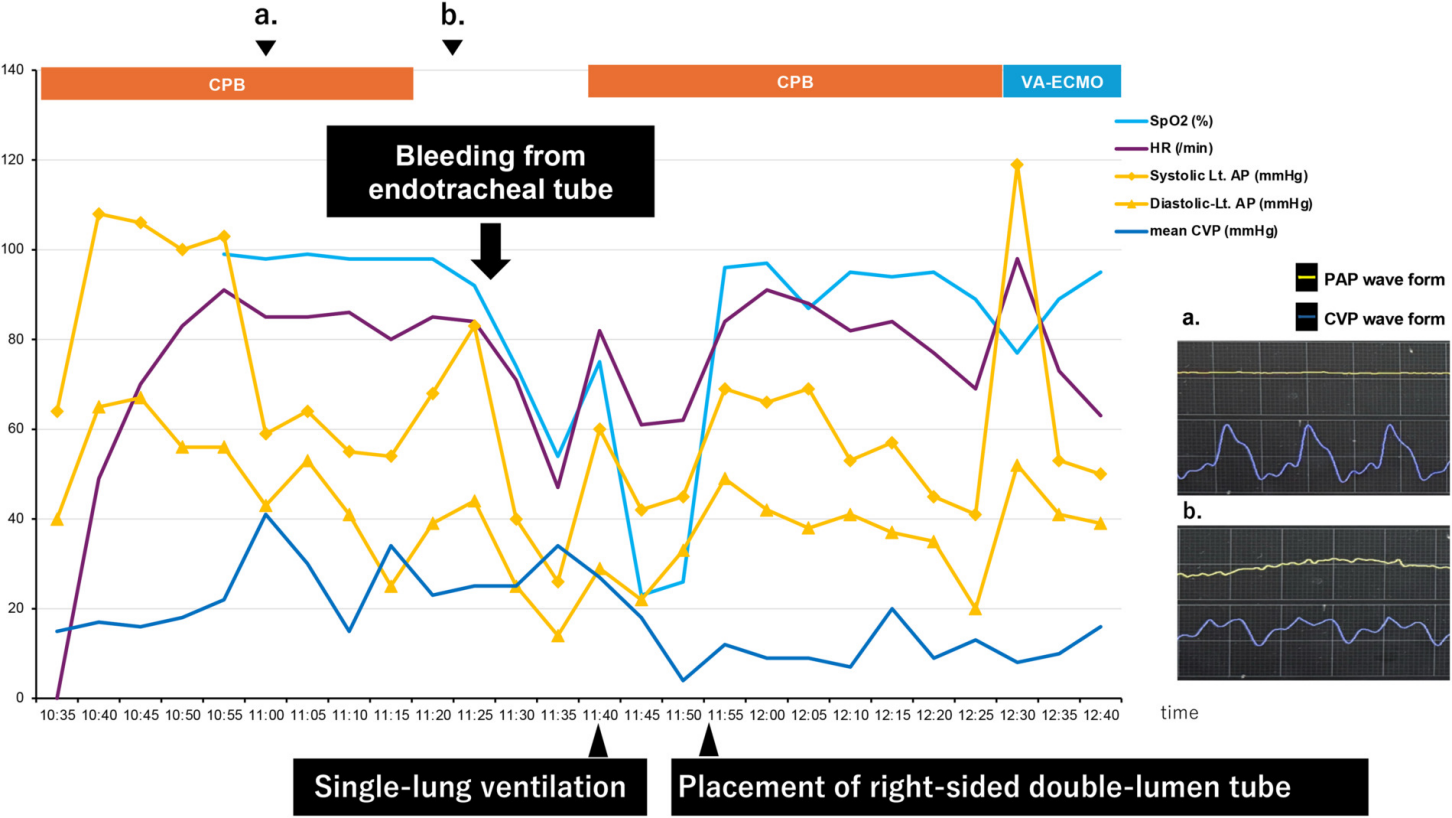
Timeline.

The timeline after occurrence of PAC-induced pulmonary artery injury is shown below (Figure 1).

### 2.2 Diagnostic assessment of event

A flat PAP waveform and a right ventricular pattern on the CVP waveform were observed prior to weaning from CPB, as shown in the timeline. The catheter depth migrated deeper from 55 to 56 cm. The PAC was retracted to 30 cm with the balloon deflated and readjusted, but the flat PAP waveform remained unchanged. After CPB weaning, a gradual decrease in peripheral oxygen saturation (SpO_2_) was observed immediately before protamine administration, although other vital signs remained stable. During protamine infusion, pulsatile bleeding from the endotracheal tube (ETT) was noted. Even after ETT suctioning, hemostasis could not be achieved, and the patient's blood pressure began to fall. CPB was re-established to provide hemodynamic support and regulate pulmonary artery blood flow.

The surgeons checked the surgical site, the lungs, and the peri-airway area at our request, but no abnormalities were found. The ETT was blindly advanced deeper into the trachea to achieve single-lung intubation. Pulsatile bleeding continued within the ETT alone, with no oral bleeding. Withdrawal of the ETT under suction confirmed that the bleeding originated from the right bronchus. Based on these clinical findings, pulmonary artery injury was strongly suspected as the source of hemorrhage.

### 2.3 Interventions

The single-lumen ETT was replaced with a 37-Fr right-sided double-lumen tube (DLT), positioned distally in the right upper lobe bronchus. Only the right bronchial lumen of the DLT was clamped, achieving compressive hemostasis and tamponade of the bleeding sites in the right middle and lower lobes. Adequate ventilation was subsequently achieved, and the vital signs stabilized. To prevent alveolar collapse in the unaffected lung while oxygenation and ventilation were supported by CPB, PEEP was maintained at 10 cmH₂O and recruitment maneuvers were applied using an airway pressure of 30 cmH₂O sustained for 10 s. The patient was switched from CPB to venoarterial extracorporeal membrane oxygenation (VA-ECMO) for transfer to the intensive care unit (ICU). A small dose of protamine was administered to maintain the activated clotting time (ACT) between 150 and 200 s. Coagulation was further optimized with transfusions of platelets and fresh frozen plasma, along with careful regulation of body temperature and calcium levels. Total anesthesia time was 7 h and 8 min; surgical duration was 5 h and 50 min; and estimated blood loss was 5,815 ml, including approximately 3,000 ml from ETT bleeding. Postoperative coagulation status was corrected with transfusions, electrolytes, and coagulation factors. Hemostasis was achieved within 3 h after ICU admission.

### 2.4 Follow-up and outcomes

**Figure 2:**
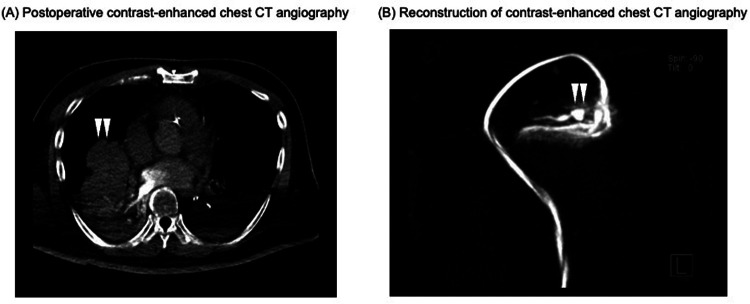
Postoperative contrast-enhanced chest CT angiography on POD 7. **(A)** Postoperative contrast-enhanced chest CT angiogram. The white arrows indicate atelectasis surrounding a pseudoaneurysm in the right middle lobe branch. **(B)** Reconstruction of contrast-enhanced chest CT angiogram at the site of the pseudoaneurysm and surrounding vessels. The white arrows indicate the site of the pseudoaneurysm. CT, computed tomography; POD, postoperative day.

**Figure 3:**
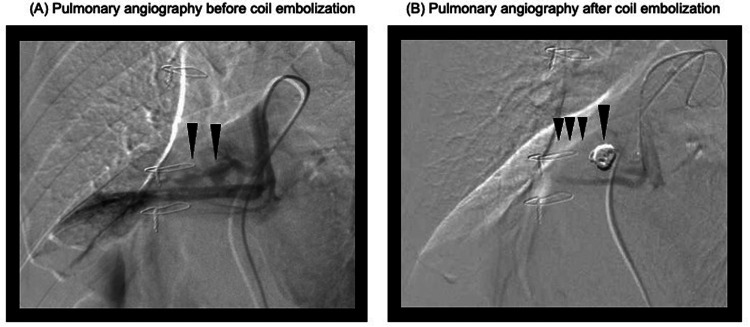
Pulmonary angiograms before and after coil embolization. **(A)** Pulmonary angiogram before coil embolization. The black arrows indicate the target pulmonary artery branch planned for embolization. **(B)** Pulmonary angiogram after coil embolization. The black arrows indicate the successfully occluded pulmonary artery branch.

Within the first postoperative day, the patient was successfully weaned off VA-ECMO with stable hemodynamics. However, intermittent decline in oxygenation was observed due to persistent minor tracheal bleeding and blood clots causing bronchial obstruction. On postoperative day 7, pulmonary artery angiography revealed a pseudoaneurysm in the right middle lobe branch and surrounding atelectasis (Figure 2). Coil embolization was performed successfully, resolving the pseudoaneurysm and preventing further bleeding (Figure 3). Following embolization, the tracheal bleeding was resolved, and the patient's respiratory condition improved gradually. A tracheostomy was required on postoperative day 11 due to prolonged intubation. No additional surgical complications were found.

## 3 Discussion

We report a case of pulmonary artery injury associated with PAC use, which resulted in intratracheal bleeding of 3,000 ml after weaning from CPB. The condition was successfully managed without the need for pulmonary resection, through comprehensive management including reinstitution of CPB to stabilize hemodynamics and limit pulmonary blood flow, placement of a right-sided double-lumen tube to compress the injured site and protect the contralateral lung, and coil embolization of a pseudoaneurysm.

Massive intratracheal bleeding during cardiac surgery is rare but carries a high risk of mortality. The causes can be categorized into three main types: underlying pulmonary disease, surgical trauma, and PAC-related injury. In this case, surgical exploration under bloodless conditions during CPB detected no surgical trauma, visceral pleural injury, and hematoma. When pulmonary blood flow was restricted, adjustment of the single-lumen ETT from a bilateral ventilation position to a single-lung ventilation position revealed active bleeding from the right lung. Prior to CPB weaning, a flattened PAP waveform and a CVP waveform indicative of right ventricular pressure suggested that the PAC had migrated too distally during CPB. These clinical findings strongly suggested PAC-induced pulmonary artery injury, particularly given that over 90% of such injuries originate from the right lung (7).

PAC-related pulmonary artery injury is a rare complication, with an incidence of 0.1%–0.8% (7). Although the extent of bleeding varies, the reported mortality rate is as high as 45%–60% (7). Risk factors include advanced age, female sex, pulmonary hypertension, hypothermia-induced catheter stiffness, over-insertion, and anticoagulation therapy (7, 8). Especially, deeper hypothermia may further increase PAC stiffness, leading to a higher risk of PAC-induced pulmonary artery injury (9). In the present case, hypothermia and over-insertion likely contributed to the complication. Furthermore, several mechanisms for PAC-induced pulmonary artery injury have been proposed, including balloon overinflation, eccentric balloon inflation caused by damage to the catheter tip, peripheral wedging of the catheter tip, and rapid distal migration with catheter tip impact leading to pulmonary artery trauma. Preventive methods include withdrawing the catheter by 3–5 cm after insertion and prior to CPB initiation, limiting balloon inflation, avoiding inflation during wedging, and positioning the PAC near the proximal pulmonary artery (8). The importance of appropriately positioning the PAC in the proximal right pulmonary artery under TEE guidance has been well discussed in previous studies. Adding pressure waveform analysis can further enhance the accuracy of catheter placement (10, 11). In the present case, the PAC was advanced into the pulmonary artery under TEE guidance, and its position was adjusted by monitoring the pressure waveform. Once the catheter reached the wedge position, it was withdrawn by 3 cm while monitoring the pressure waveform and secured at 55 cm. To ensure appropriate placement, it would have been preferable to confirm the catheter balloon's position in the proximal right pulmonary artery under TTE guidance after identifying the wedge position and gradually withdrawing the catheter. This adjustment could have also helped eliminate slack in the catheter. If slack had remained in the PAC, the slack itself might have contributed to unintended distal migration of the catheter. In such a case, the catheter could have been fixed at the superior vena cava by the venous cannulation tourniquet, and intracardiac collapse or surgical manipulation might have caused it to advance further into the pulmonary artery. Furthermore, verifying the absence of slack during initial placement using C-arm fluoroscopy, or withdrawing the PAC by approximately 3 cm after CPB initiation, might have helped prevent this advancement.

Prior to CPB weaning, the PAC was found to have migrated deeper from 55 to 56 cm, prompting an attempt to reposition it by withdrawing to 30 cm. However, proper waveforms could not be obtained. This observation suggests that the catheter tip had advanced distally during CPB, potentially causing trauma to the pulmonary artery. Examination of the PAC upon removal showed no damage, suggesting that the injury was caused by the catheter tip impacting the distal pulmonary artery wall.

Management of massive intratracheal bleeding begins with securing ventilation. Minor bleeding can be effectively managed with frequent suctioning. However, significant bleeding requires a more aggressive approach, including isolation of the injured lung and protection of the unaffected lung using a bronchial blocker or DLT. In cases of massive intratracheal bleeding, such as the 3,000 ml volume in the present case, identifying the affected lung can be challenging. Restricting pulmonary blood flow may facilitate identification of the affected lung and subsequent protection. Approximately 65% of PAC-related pulmonary artery injuries occur during CPB weaning (8), whereas symptoms appeared after CPB weaning in the present case. We reinstituted CPB to restrict pulmonary blood flow and adjusted the single-lumen ETT to provide single-lung ventilation, allowing bronchoscopic identification of the affected lung. A right-sided DLT was then positioned distal to the right upper lobe branch, achieving targeted isolation and tamponade of the injured area and simultaneously ensuring adequate ventilation. To prevent alveolar collapse in the unaffected lung while oxygenation and ventilation were supported by CPB, PEEP was maintained at 10 cmH₂O and recruitment maneuvers were applied using an airway pressure of 30 cmH₂O sustained for 10 s.

While emergency pulmonary resection is an option for uncontrolled massive intratracheal bleeding, the procedure is associated with increased mortality during cardiac surgery (7). After consultation with the thoracic surgery team, we determined that resection was not warranted, as there was no evidence of diffuse parenchymal hemorrhage, visceral pleural rupture, or central pulmonary artery damage. Active bleeding subsided within three hours postoperatively, permitting bilateral lung ventilation and VA-ECMO removal on postoperative day 1. Postoperative rebleeding is reported in 45% of cases within 48 h to 14 days. In the present case, despite frequent suctioning and hemostatic management, minor hemoptysis persisted, and intermittent hypoxemia occurred during position changes. Pulmonary angiography on postoperative day 7 revealed a pseudoaneurysm in the right middle lobe branch and surrounding atelectasis, which was successfully treated with coil embolization.

We conducted a literature search and reviewed all reported cases of PAC-induced pulmonary artery injury published between 1990 and 2024 (Table 1) (12–22). A comprehensive search on PubMed® used the keywords “pulmonary artery catheter,” “Swan-Ganz catheter,” “cardiac surgery,” “complications,” “pulmonary artery rupture,” “pulmonary artery injury,” “intrapulmonary artery injury,” “vascular perforation,” and “hemorrhage”. All the identified review articles, randomized-control trials and metanalysis were hand-searched for additional references, and reference lists for identified studies were snowballed for additional articles (1, 2, 23, 24). As a result, 23 articles were identified. After excluding duplicates, non-English publications, and inaccessible full-text articles, 11 articles documenting 21 cases were reviewed. The patient population consisted of 16 females aged between 67 and 84 years (mean: 76 years) and 5 males aged between 55 and 77 years (mean: 67 years). The surgeries comprised 11 valve replacements and 10 CABG procedures. Hemoptysis was the initial sign of injury in 86% of the cases, and most pseudoaneurysms (90%) were located in the right pulmonary artery. Coil embolization was the most common treatment (48%), with surgical intervention required in one case. Mortality was reported in two cases. The present case uniquely occurred after CPB weaning, whereas most injuries in the review occurred during CPB weaning (33%). Furthermore, although the literature review identified only two cases in which MCS was used, this case highlights the effectiveness of pulmonary blood flow restriction via CPB resumption in controlling massive intratracheal bleeding and facilitating the identification of the injury site. These findings highlight the unique aspects of this case report and underscore the importance of vigilance throughout the perioperative period and the potential role of MCS in managing PAC-induced pulmonary artery injury.

**Table 1:** Demographics, clinical course, and outcome of the reviewed cases.

First author (year)	No. of cases	Age and sex	Risk factors*	Indications for PAC	Onset timing of PA injury symptoms	MCS device use	Initial evidence of PA injury	Duration between PAC insertion and initial evidence of PA injury (days)	Site of pseudo-aneurysm	Treatment	Outcome	References No.
De Lima (1994)	1	76F	1,2,3	MVR	On weaning from CPB	-	Hemoptysis	0	RML	No treatment	Discharged	(8)
Cicenia (1996)	1	69F	1,2	CABG	No trigger	-	Hemoptysis	0	RUL	Coil embolization	Discharged	(9)
Feritti (1996)	5	69M	NA	CABG	NA	-	Hemoptysis	5	RLL	Coil embolization	Discharged	(10)
		74F	NA	CABG	NA	-	Hemoptysis	19	RML	Coil embolization	Discharged	(10)
		75F	NA	MVR	NA	-	Hemoptysis	0	RML	Coil embolization	Discharged	(10)
		81F	NA	CABG	NA	-	Hemoptysis	0	RML	Coil embolization	Discharged	(10)
		67F	NA	CABG	NA	-	Hemoptysis	2	RLL	Coil embolization	Discharged	(10)
Mullerworth (1998)	3	83F	NA	CABG	On weaning from CPB	-	Hemoptysis	0	NA	No treatment	Discharged	(11)
		84F	NA	AVR	Continuous wedged	-	Hemoptysis	2	NA	NA	Died from exsanguination	(11)
		71F	NA	CABG	On weaning from CPB	ECMO	Hemoptysis, hypotension, hypoxemic	0	RML	PAC withdrawn only	Discharged	(11)
Laureys (2004)	1	75F	1,2,4	AVR + MVR	Thoracic closure	-	Hypotension, CXR	0	RML	Coil embolization	Discharged	(12)
Sidery (2004)	1	71F	1,2,4	CABG	No trigger	-	CXR	10	RLL	No treatment	Discharged	(13)
Bossert (2006)	2	64M	no	CABG	PCWP measurement	-	Hemoptysis	0	RLL	Intraluminal vasopressin and assisted ventilation with PEEP of 15 cm H_2_O	Discharged	(14)
		80F	1,2	CABG	NA	-	VF, Hemoptysis	0	RML	Lobectomy	Discharged	(14)
Bianchini (2007)	1	75F	1,2	AVR + MVR	After weaning from CPB	ECMO/IABP	Hypotension, Hypoxia, Hemoptysis	0	RLL	Coil embolization	Discharged	(15)
Burrel (2010)	3	77M	no	AVR	No trigger	-	Hemoptysis	1	RML	Plug	Discharged	(16)
		75F	1,2,3,4	AVR + MVR	No trigger	-	Hemoptysis	14	RML	Plug	Died from cerebral hematoma	(16)
		77F	1,2,3	AVR + MVR	No trigger	-	Hemoptysis	1	RML	Plug	Discharged	(16)
Rudziński (2016)	2	55M	NA	AVR	No trigger	-	Hemoptysis	7	RLL	Coil embolization	Discharged	(17)
		68M	NA	AVR	No trigger	-	CXR	0	RLL	Plug	Discharged	(17)
Russo (2024)	1	82F	1,2	MVR + TVR	Balloon deflation		Hemoptysis	0	RML	Coil embolization	Discharged	(18)

MVR, mitral valve replacement; CABG, coronary artery bypass graft; AVR, aortic valve replacement; TVR, tricuspid valve replacement; CPB, cardiopulmonary bypass; NA, data not applicable; ECMO, extracorporeal membrane oxygenation; IABP, intra-aortic balloon pumping; CXR, chest x-ray; VF, ventricular fibrillation; RML, right middle lobe; RUL, right upper lobe; RLL, right lower lobe; PAC, pulmonary artery catheter; PEEP, Positive End-Expiratory Pressure.

* Risk factors: 1. age > 60 years, 2. female sex, 3. pulmonary hypertension, 4. systemic anticoagulation, 5. long-term steroid use, and 6. surgically induced hypothermia.

## 4 Conclusion

We report a case of massive intratracheal bleeding caused by PAC-induced pulmonary artery injury, which was successfully managed through comprehensive treatment without the need for pulmonary resection. This case provides two important lessons. First, restricting pulmonary blood flow during massive intratracheal bleeding is effective not only for stabilizing hemodynamic but also for identifying the cause and site of injury. Second, precise localization and control of the injured area combined with a multidisciplinary approach to comprehensive management can avoid pulmonary resection and achieve successful outcomes. We hope this report serves as a valuable reference for the management of similar complications in the future.

## Data Availability

The original contributions presented in the study are included in the article/Supplementary Material, further inquiries can be directed to the corresponding author.

## References

[1] Binanay C Califf RM Hasselblad V O’Connor CM Shah MR Sopko G Evaluation study of congestive heart failure and pulmonary artery catheterization effectiveness: the ESCAPE trial. JAMA. (2005) 294:1625–33. 10.1001/jama.294.13.1625 16204662

[2] Sandham JD Hull RD Brant RF Knox L Pineo GF Doig CJ A randomized, controlled trial of the use of pulmonary-artery catheters in high-risk surgical patients. N Engl J Med. (2003) 348:5–14. 10.1056/NEJMoa021108 12510037

[3] Garan AR Kanwar M Thayer KL Whitehead E Zweck E Hernandez-Montfort J Complete hemodynamic profiling with pulmonary artery catheters in cardiogenic shock is associated with lower in-hospital mortality. JACC Heart Failure. (2020) 8:903–13. 10.1016/j.jchf.2020.08.012 33121702

[4] Judge O Ji F Fleming N Liu H. Current use of the pulmonary artery catheter in cardiac surgery: a survey study. J Cardiothorac Vasc Anesth. (2015) 29:69–75. 10.1053/j.jvca.2014.07.016 25440650

[5] Brovman EY Gabriel RA Dutton RP Urman RD. Pulmonary artery catheter use during cardiac surgery in the United States, 2010 to 2014. J Cardiothorac Vasc Anesth. (2016) 30:579–84. 10.1053/j.jvca.2015.11.012 26947712

[6] Joseph C Garrubba M Smith JA Melder A. Does the use of a pulmonary artery catheter make a difference during or after cardiac surgery? Heart Lung Circ. (2018) 27:952–60. 10.1016/j.hlc.2018.02.004 29555415

[7] Urschel JD Myerowitz PD. Catheter induced pulmonary artery rupture in the setting of cardiopulmonary bypass. Ann Thorac Surg. (1993) 56:585–9. 10.1016/0003-4975(93)90912-2 8379744

[8] Kearney TJ Shabot MM. Pulmonary artery rupture associated with the swan-ganz catheter. Chest. (1995) 108:1349–52. 10.1378/chest.108.5.1349 7587440

[9] Cohen JA Blackshear RH Gravenstein N Woeste J. Increased pulmonary artery perforating potential of pulmonary artery catheters during hypothermia. J Cardiothorac Vasc Anesth. (1991) 5:234–6. 10.1016/1053-0770(91)90280-7 1863743

[10] Cronin B Robbins R Maus T. Pulmonary artery catheter placement using transesophageal echocardiography. J Cardiothorac Vasc Anesth. (2017) 31:178–83. 10.1053/j.jvca.2016.07.012 27707512

[11] Cronin B Kolotiniuk N Youssefzadeh K Newhouse B Schmidt U O’Brien EO Pulmonary artery catheter placement aided by transesophageal echocardiography versus pressure waveform transduction. J Cardiothorac Vasc Anesth. (2018) 32:2578–82. 10.1053/j.jvca.2018.05.018 29929894

[12] DeLima LG Wynands JE Bourke ME Walley VM. Catheter-induced pulmonary artery false aneurysm and rupture: case report and review. J Cardiothorac Vasc Anesth. (1994) 8:70–5. 10.1016/1053-0770(94)90016-7 8167290

[13] Cicenia J Shapira N Jones M. Massive hemoptysis after coronary artery bypass grafting. Chest. (1996) 109:267–70. 10.1378/chest.109.1.267 8549196

[14] Ferretti GR Thony F Link KM Durand M Wollschläger K Blin D False aneurysm of the pulmonary artery induced by a swan-ganz catheter: clinical presentation and radiologic management. AJR Am J Roentgenol. (1996) 167:941–5. 10.2214/ajr.167.4.8819388 8819388

[15] Mullerworth MH Angelopoulos P Couyant MA Horton AM Robinson SM Petring OU Recognition and management of catheter-induced pulmonary artery rupture. Ann Thorac Surg. (1998) 66:1242–5. 10.1016/s0003-4975(98)00593-1 9800813

[16] Laureys M Golzarian J Antoine M Desmet JM. Coil embolization treatment for perioperative pulmonary artery rupture related to swan-ganz catheter placement. Cardiovasc Intervent Radiol. (2004) 27:407–9. 10.1007/s00270-004-0164-8 15129332

[17] Sidery M Cahir J Screaton NJ Vuylsteke A. Computed tomography reveals an unusual complication in a patient having undergone coronary artery bypass surgery. J Cardiothorac Vasc Anesth. (2004) 18:668–70. 10.1053/j.jvca.2004.07.020 15578485

[18] Bossert T Gummert JF Bittner HB Barten M Walther T Falk V Swan-Ganz catheter-induced severe complications in cardiac surgery: right ventricular perforation, knotting, and rupture of a pulmonary artery. J Card Surg. (2006) 21:292–5. 10.1111/j.1540-8191.2006.00235.x 16684066

[19] Bianchini R Melina G Benedetto U Rossi M Fiorani B Iasenzaniro M Extracorporeal membrane oxygenation for Swan-Ganz induced intraoperative hemorrhage. Ann Thorac Surg. (2007) 83:2213–4. 10.1016/j.athoracsur.2007.01.023 17532432

[20] Burrel M Real MI Barrufet M Arguis P Sánchez M Berrocal L Pulmonary artery pseudoaneurysm after swan-ganz catheter placement: embolization with vascular plugs. J Vasc Interv Radiol. (2010) 21:577–81. 10.1016/j.jvir.2009.12.399 20207165

[21] Rudziński PN Henzel J Dzielińska Z Lubiszewska BM Michałowska I Szymański P Pulmonary artery rupture as a complication of swan-ganz catheter application. Diagnosis and endovascular treatment: a single centre’s experience. Postepy Kardiol Interwencyjnej. (2016) 12:135–9. 10.5114/aic.2016.59364 27279873 PMC4882386

[22] Russo R Calzolari A Salice V Micieli C Castiglioni C Gomarasca M Pulmonary artery pseudoaneurysm due to pulmonary artery catheter placement: a new minimally invasive approach to solve a life-threatening complication. J Cardiothorac Vasc Anesth. (2025) 39:215–9. 10.1053/j.jvca.2024.10.017 39505579

[23] Rong LQ Luhmann G Di Franco A Dimagli A Perry LA Martinez AP Pulmonary artery catheter use and in-hospital outcomes in cardiac surgery: a systematic review and meta-analysis. Interdiscip Cardiovasc Thorac Surg. (2024) 39:ivae129. 10.1093/icvts/ivae129 38976638 PMC11254303

[24] Nellaiyappan M Omar HR Justiz R Sprenker C Camporesi EM Mangar D. Pulmonary artery pseudoaneurysm after swan-ganz catheterization: a case presentation and review of literature. Eur Heart J Acute Cardiovasc Care. (2014) 3:281–8. 10.1177/2048872613520252 24470440

